# A Polymer-Based Indicator for Detecting Dexamethasone in Herbal Medicine Using Polymethylmethacrylate (PMMA)

**DOI:** 10.3390/polym15132862

**Published:** 2023-06-28

**Authors:** Rimadani Pratiwi, Vandie Charlie, Nyi Mekar Saptarini, Driyanti Rahayu

**Affiliations:** Department of Pharmaceutical Analysis and Medicinal Chemistry, Faculty of Pharmacy, Universitas Padjadjran, Bandung 45363, Indonesia; vandiecharlie1@gmail.com (V.C.); nyi.mekar@unpad.ac.id (N.M.S.); driyanti.rahayu@unpad.ac.id (D.R.)

**Keywords:** dexamethasone, polymethylmethacrylate (PMMA), indicator strip

## Abstract

Dexamethasone is a chemical drug that is usually added to herbal medicine because of its effects on pain relief, arthritis, anti-inflammation, etc. Chemical drugs should not be used in herbal medicine because uncontrolled consumption causes some side effects. A polymer-based indicator was developed to detect dexamethasone in herbal medicine samples in a fast and simple way compared to instrumental analysis. The indicator strips were made by mixing polymethylmethacrylate (PMMA) polymer with sulfuric acid (H_2_SO_4_) as a reagent. When reacting with dexamethasone, H_2_SO_4_ can cause the color to change into a specific light pink-purple color. Indicator strips were prepared with a composition of 5% PMMA in ethyl acetate:H_2_SO_4_ (9:1) by using the reagent blending method. The indicator strips showed a pink-purple color when they were applied to a positive herb containing dexamethasone. The indicator strips could selectively detect dexamethasone rather than other active substances that are often found in herbal medicine. These indicator strips could also detect dexamethasone with the smallest detection limit of 13.13 ppm, and they had a stability of up to 36 days. Detection was carried out in real samples to show the performance of the indicator strips. The result showed that of nine samples, five were confirmed to contain dexamethasone. These results showed a good agreement with the results of thin-layer chromatography (TLC) and high-performance liquid chromatography (HPLC). According to the result, these indicator strips provide a simple and applicable method for on-site analysis to detect dexamethasone in samples.

## 1. Introduction

Herbal medicines are natural plant-derived substances that contain phytochemical compounds that are used for medicinal or treatment purposes [1]. Herbal medicines are still widely used to empirically treat or prevent diseases [2,3]. According to the Ministry of Industry in Indonesia, in 2017, there were 986 herbal medicine industries, of which 102 were traditional drug industries, and the rest included small traditional medicine businesses that were spread across Indonesia [4]. Regulation Number 007 of 2012 Article 7 from the Minister of Health of the Republic of Indonesia states that traditional medicines are not allowed to contain chemical drugs [5]. Due to the potential market in Indonesia, some herbalists often add chemical drugs to herbal preparations to increase the therapeutic effects. Herbal medicines can be consumed without a doctor’s prescription, but when these herbs contain chemical drugs, the consumption of the chemical drugs in these herbs can become uncontrolled, which can increase their side effects. Between July 2020 and September 2021, the Indonesian Food and Drug Supervisory Agency found that there were at least 256 herbal products containing chemical drugs, and 21 of them contained dexamethasone, which was claimed to be an herbal medicine for aches, gout, and pain [5,6,7].

Dexamethasone is a glucocorticoid drug that has an anti-inflammatory effect, but if it is taken continuously for a long time, it causes side effects, such as diabetes, osteoporosis, Cushing syndrome, peptic ulcers, and, if given to children, impaired adrenal growth [8,9]. In addition, the uncontrolled consumption of dexamethasone can cause some adverse effects such as sleep problems, mood changes, indigestion, and weight gain. Dexamethasone is also registered as category C for pregnant women by the FDA, so herbs that contain dexamethasone and that are consumed massively and without control are very dangerous for pregnant women [10].

Dexamethasone can be detected in herbal medicine by using standardized methods of high-performance liquid chromatography (HPLC) with the procedures listed in the pharmacopeia [11]. Another method that has been developed to detect dexamethasone in herbal medicine is using UV-Vis spectrophotometry by firstly forming a dexamethasone–hydroxylamine complex so that it can be detected with a UV-Vis spectrophotometer, but the formation of this complex requires a quite complicated procedure before dexamethasone can be detected via UV-Vis spectrophotometry [12]. Dexamethasone can also be detected by using a simpler method—thin-layer chromatography (TLC)–densitometry—as described by Permatasari et al. Asra et al. conducted research by using the high-performance thin-layer chromatography (HPTLC)–densitometry method as an advanced form of TLC, which offered better resolution and faster detection than TLC due to the use of silica gel with a very small size compared to that used in TLC [13,14]. Tests for dexamethasone with the methods that were mentioned before are dependent on instruments that are quite complex in terms of use, involve complex sample preparation, are quite expensive, and cannot be performed on site. A simple and easy testing method that can be run at a low cost with no need for skilled personnel and that can be carried out on site is needed. The indicator strip method was introduced to simplify the screening process for herbal medicine containing dexamethasone and as a prospective method for on-site analysis [15,16,17].

In this study, a polymer-based indicator for dexamethasone detection in herbs was developed by using polymethylmethacrylate (PMMA) as the base material. PMMA is a good material for use in indicator strips due to its properties of excellent optical clarity, light weight, weather resistance, high strength, minimal toxicity, and good reagent retention. Indicator strips made with PMMA could show color changes that were easy to observe and had good stability [18,19,20]. PMMA was mixed with an H_2_SO_4_ reagent to cause a color change when dexamethasone was contained in the sample. The performance of this indicator was also evaluated, and the results were compared with those of HPLC.

## 2. Materials and Methods

### 2.1. Materials and Instruments

All chemicals used were of analytical grade and were directly used without further purification. Sulfuric acid (H_2_SO_4_) and chloroform were obtained from Merck. HPLC-grade acetonitrile and methanol were purchased from J.T. Baker. Acetone was obtained from Mallinckrodt Chemicals. Ethyl acetate, standard dexamethasone, standard paracetamol, standard allopurinol, standard antalgin, standard phenylbutazone, standard mefenamic acid, and standard chlorpheniramine maleate (CTM) were obtained from Sigma-Aldrich. PMMA and TLC silica gel plates (GF254) were obtained from Merck. The chromatographic measurements were carried out by using high-performance liquid chromatography with a Shimadzu HPLC SPD-10Avp series instrument.

### 2.2. Sample Collection and Preparation

The herbal samples used were herbs with claims of relieving pain, gout, and rheumatic aches. Ten products of herbal medicine were collected from various offline and online stores. The samples were checked for their registration numbers on the webpage of the Indonesian Food and Drug Administration (https://cekbpom.pom.go.id/ (accessed on 23 January 2023)) [21]. Nine of the ten products were used as test samples, while one product was used as a control. The products that were used as negative controls were products that did not contain dexamethasone according to their registration on the Indonesian FDA website, TLC, or HPLC. For the positive control, a dexamethasone standard was added to the negative control. The samples were prepared by extracting a total of one dose per drink based on each sample with 30 mL of chloroform:methanol (9:1). Then, they were stirred with a shaker at 90 rpm for 30 min. This solvent combination was used in previous studies to detect dexamethasone and showed good results, so the same combination was also used to extract dexamethasone in the herbal samples here [12]. The stirring process also served to ensure that the dexamethasone in the herbal samples could be optimally extracted. The samples were then centrifuged at 4000 rpm for 5 min to separate the dissolved and insoluble substances from the sample. The supernatant was taken and evaporated in a water bath at 70 °C until dry. The residue was then dissolved with 5 mL of methanol, and the solution was filtered and stored for further testing [22,23]. This preparation was also performed for the negative control and positive control. The reproducibility of the extraction process was determined by using the positive control, and the results were directly measured by using HPLC. The results showed that the %recovery of the extraction was 75.27% ± 0.18%.

### 2.3. Selection of the Reagent for Dexamethasone Detection

The reagent used in this study was a reagent that could show a color change when it reacted with dexamethasone. Sulfuric acid (H_2_SO_4_) can react with dexamethasone in the form of a solid or a solution to produce a light pink-purple color [24]. Therefore, H_2_SO_4_ was chosen as a reagent for dexamethasone detection.

### 2.4. Fabrication of PMMA-Based Indicator Strips

PMMA indicator strips were prepared with the blending method by dissolving PMMA in ethyl acetate and the reagent until a 5% PMMA concentration was reached. Initially, PMMA was dissolved with ethyl acetate and then stirred with a magnetic stirrer at a speed of 250 rpm until the polymer dissolved. H_2_SO_4_ was then added to the solution while stirring for 5–10 s to homogenize the reagent with the PMMA solution [25]. After becoming homogeneous, the indicator solution was transferred to a Petri dish and dried in a desiccator for 24 h until a film base was formed. The film base was collected in a pot and was then ready to use as an indicator.

### 2.5. Performance Test

To ensure the quality of the indicator strips, performance tests, including tests of selectivity, accuracy, sensitivity, and stability, were carried out [26].

#### 2.5.1. Selectivity Test

The selectivity test was conducted to ensure that the reagent could detect only dexamethasone and not other active substances in the sample. The test was carried out by comparing the results of the reaction between the reagent with dexamethasone and that with other chemical drugs that are also often found in herbal medicine, such as paracetamol, allopurinol, antalgin, phenylbutazone, mefenamic acid, and chlorpheniramine maleate (CTM). These chemical drugs were dissolved in a solvent suitable for the active substance being tested, and they were tested as comparison substances that were exposed to reagent. The expected result was a difference in color between the reagent exposed to dexamethasone and the reagent exposed to the other active substances used for the comparison [27].

#### 2.5.2. Accuracy Test 

An accuracy test was carried out to ensure that the performance of the polymer-based indicator was comparable with that of thin-layer chromatography (TLC) and high-performance liquid chromatography (HPLC). 

The prepared samples were first screened with TLC by spotting each sample onto a silica gel GF 254 plate for a comparison of the dexamethasone standard and the positive and negative controls. The TLC plate was eluted with the mobile phase of chloroform:acetone (4:1). Then, the retention factor (Rf) value of each sample was calculated and compared with that of the dexamethasone standard. If the Rf value of the sample was similar to the Rf of the dexamethasone standard, the sample was positive for dexamethasone. The positive samples containing dexamethasone according to TLC were further analyzed with HPLC and the indicator strips to confirm the results [28,29].

The test was carried out with a Shimadzu LC-10AT HPLC instrument with a Zorbax HPLC Column Eclipse XDB-C18 (15 cm). The mobile phase ratio used was acetonitrile:water (30:70) with a 0.8 mL/min flow rate, and detection was carried out at a wavelength of 240 nm. To determine the concentration of dexamethasone in herbal medicine, a calibration curve was prepared from concentrations of 1, 5, 10, 20, 30, 40, 60, 100, and 150 ppm. The standard solution and sample were filtered with a 0.45 μm filter membrane before being analyzed with HPLC. The concentration of dexamethasone in the sample was calculated based on the linearity that was obtained [30]. The indicator strip was made by using filter paper, and a positive sample containing dexamethasone according to TLC was exposed to the indicator strip. The results of the color change were observed.

#### 2.5.3. Sensitivity Test

A sensitivity test was carried out by diluting the herb samples that were positive for dexamethasone with dilution factors (DF) of 0, 10, 20, and 40. The color change for each DF was observed, and the last DF at which the indicator strip showed a visually perceptible color change was determined. The higher the DF, the better the sensitivity because the concentration detected was smaller, so the indicator strip could detect dexamethasone with smaller concentrations in herbs.

#### 2.5.4. Stability Test

A stability test was carried out on the indicator film base in a pot stored in a plastic container filled with silica gel. Tests were carried out every day by making indicator strips and exposing them to herbal samples until the strips did not show the appropriate color change. The longer the indicator strips could detect dexamethasone in herbal medicine, the better the quality of the indicator strips, which meant that these indicator strips could be stored under controlled conditions and then be used again [31].

## 3. Results and Discussion

### 3.1. Sample Collection and Preparation

Ten products of herbal medicine with claims of relieving pain, gout, and rheumatic aches were collected from various herbal medicine shops in Indonesia. The sample collection process was carried out by buying samples from offline and online stores. Nine of the ten products were used as the test samples, while one product was used as a control. All products were checked for their legality on the website of the Food and Drug Administration of Indonesia. Among the ten herbal products, three legal samples and seven illegal samples were detected, as shown in Table 1. Two of the seven illegal samples (S4 and S9) were included in the public warning list by the Indonesian Food and Drug Monitoring Agency [7]. 

All samples were extracted by using chloroform:methanol (9:1) before the analysis was continued. This solvent ratio was considered to be able to properly extract dexamethasone from the herbal samples so that dexamethasone could be better detected [32]. The dried residue that was obtained from the drying process after using a water bath was then dissolved with 5 mL of methanol and filtered with filter paper to remove insoluble particles. Then, the solution was stored in bottles for further testing.

### 3.2. Selection of the Reagent for Dexamethasone Detection

**Figure 1:**
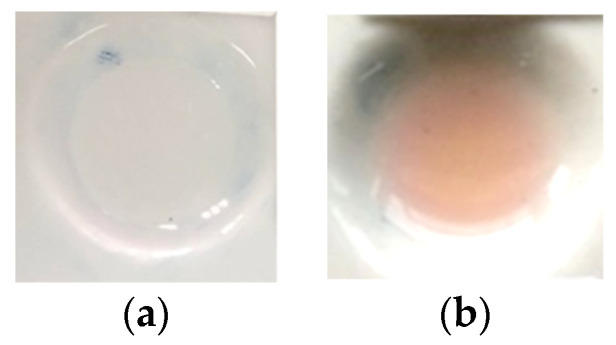
(**a**) Blank of dexamethasone before the addition of H_2_SO_4_; (**b**) color change of dexamethasone after the addition of H_2_SO_4_.

A color change occurred when H_2_SO_4_ was exposed to standard dexamethasone, producing a light pink-purple color, as seen in Figure 1b, which was different from the color of dexamethasone prior to its exposure to sulfuric acid, as shown in Figure 1a [24]. This color change made it possible to visually observe the detection of dexamethasone. In addition, H_2_SO_4_ could also be combined with PMMA, so the combination of PMMA and H_2_SO_4_ as the reagent could be formed and used as an indicator strip to detect dexamethasone in herbal samples.

### 3.3. Fabrication of the PMMA-Based Indicator Strips

The PMMA-based indicator strips were made with a concentration of 5% because that concentration resulted in a strong indicator strip with less dense pores [25]. The solvent used was ethyl acetate:H_2_SO_4_ (4:1), which was obtained with the reagent blending method. The reagent was mixed with the solvent so that the reagent would absorb the polymer. The solvent ratio was 4:1, which is the optimal ratio for forming indicator strips with PMMA and an H_2_SO_4_ reagent. More H_2_SO_4_ would make the strip difficult to dry; therefore, this ratio was used for fabrication. 

**Figure 2:**
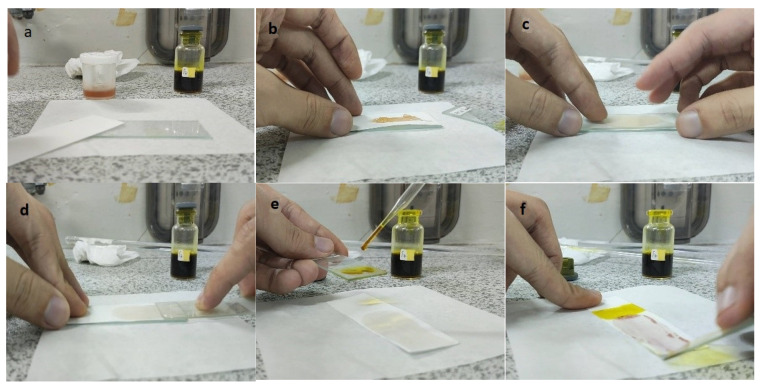
Process of making the PMMA-based indicator. (**a**) Preparation, (**b**) placing the indicator film base, (**c**) pressing the indicator film base, (**d**) removing the filter paper with a glass object, (**e**) dropping the sample, and (**f**) testing the sample on the indicator strip.

The steps of the application of the PMMA-based indicator are shown in Figure 2. To use the indicator, the film base first needed to be made into indicator strips by taking some of the indicator film base that was collected in a pot and then placing it on filter paper with a size of 2.5 cm × 7.5 cm to match the size of the glass object that was used as a base and shaper, as shown in Figure 2a,b. The larger the surface area of the filter paper, the more visible the color change would be. After that, to form the indicator strips, another glass object was used. This was placed on top of the filter paper and then pressed until a PMMA indicator strip was formed on the thin filter paper, but it had a larger surface area (Figure 2c). This was intended to increase the contact of the indicator strip with the sample and to produce a thin indicator strip. The upper part of the glass object was removed by pulling it so that the indicator strip that was formed could be exposed to the sample (Figure 2d). For testing, the sample was first dripped onto another glass object that held 2–3 drops of the herbal sample. Then, the glass object with the sample was exposed to the PMMA indicator strip by using a smearing or swiping method so that the PMMA indicator strip would be fully exposed to the sample (Figure 2e). A positive result was observed when there was a change in the indicator strip’s color to pink-purple, which indicated that the herbal sample contained dexamethasone. 

### 3.4. Performance Test

#### 3.4.1. Selectivity Test

The selectivity test was carried out with chemical drugs other than dexamethasone that are often found in herbal medicine, such as paracetamol, allopurinol, antalgin, phenylbutazone, mefenamic acid, and chlorpheniramine maleate (CTM). These chemical drugs are also added to herbal products that claim to alleviate rheumatic aches. Sometimes, these drugs are combined with dexamethasone or used as the single active substance in herbal products that are indicated as containing chemical drugs in a public warning by the Indonesian Food and Drug Supervisory Agency [7].

After dissolving the different active substances, the active substance solutions that were used for comparison were then exposed to the H_2_SO_4_ reagent and visually observed. The results in Table 2 show that sulfuric acid underwent different color changes when exposed to the different substances, and only dexamethasone resulted in a light pink-purple color. Based on the results, sulfuric acid was selective in detecting dexamethasone.

#### 3.4.2. Accuracy Test 

**Figure 3:**
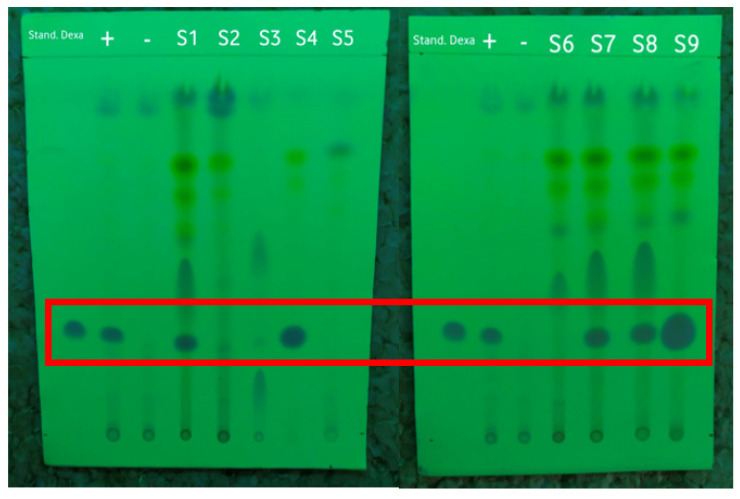
The results of screening the samples by using the TLC method under 254 nm UV light with the dexamethasone standard, positive control (+), and negative control (−) marked in the red frame.

An accuracy test was carried out to ensure the accuracy of the dexamethasone detection method by using a PMMA-based indicator in comparison with the TLC and HPLC methods. These methods have been accepted by the Indonesian Pharmacopoeia for detecting dexamethasone, so they were also used as a reference for testing the accuracy. The TLC method was used to qualitatively confirm the presence of dexamethasone in the samples. The Rf value was observed under 254 nm UV light to be able to see the dexamethasone spots, as shown in Figure 3. The Rf values of the dexamethasone standard, the positive and negative controls, and the samples can be seen in Table 3. In this test, the dexamethasone standard was used as a marker for the TLC results. The positive control of the herbal medicine was a negative control that was known not to contain dexamethasone, but standard dexamethasone was added before the extraction process. 

Based on the results shown in Table 3, the Rf value of the dexamethasone standard was 0.3. Of the nine samples of herbal medicine that were collected and prepared, five samples had Rf values similar to that of the dexamethasone standard, namely, S1, S4, S7, S8, and S9. All samples that tested positive for dexamethasone based on TLC were illegal samples; of these, S4 and S9 were samples with public warnings, while S1, S7, and S8 were unregistered samples according to the Indonesian FDA [7].

The five dexamethasone-containing samples were submitted for further analysis with the HPLC method to quantitatively confirm their content of dexamethasone. The optimization of the HPLC conditions showed good performance when the mobile-phase composition used was acetonitrile:water (30:70) with a flow rate of 0.8 mL/min and a running time of 8 min. These conditions indicated that there was a peak of dexamethasone at a retention time of 3.501 min with the UV detector at a wavelength of 240 nm, as shown in Figure S1. The conditions were optimized by fulfilling the parameters of the validation method, so the results of analyses using these conditions would show a proper detection of dexamethasone [12]. To be able to determine the level of dexamethasone in the sample, it was necessary to prepare a standard curve by using the dexamethasone standard in various concentrations. Then, the same procedure as that of the sample preparation was carried out, namely, by dissolving the dexamethasone standard with methanol and then diluting it to the required concentration. All solutions were then filtered with a 0.45 nm filter membrane and tested under the conditions described. The results of the test of each concentration were plotted on a graph. The linearity of the standard curve was y = 55,611x + 84,479 with R2 = 0.9994, and this equation was used to calculate the dexamethasone concentrations in the herbal samples, as shown in Table 4. A chromatogram of the tested samples can be seen in Figure S2 of the Supplementary Information.

The samples that were identified as positive in the TLC and HPLC analyses were then further analyzed by using a PMMA-based indicator. Positive results were indicated by a change in the color of the indicator strip to pink-purple, as shown in Table 5. Based on the results, it could be seen that all samples that were positive according to TLC and HPLC also showed positive results with the indicator strip. This indicated that the indicator strip could work well in detecting dexamethasone in herbal medicines.

#### 3.4.3. Sensitivity Test

**Figure 4:**
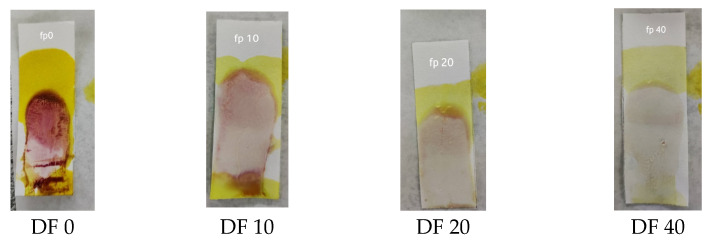
Results of the sensitivity test of the PMMA indicator strip.

A sensitivity test was carried out to determine the limit of dexamethasone concentration in herbs that could still be detected with an indicator strip. This test was carried out by directly using sample 7 (S7) with dilution because S7 had the lowest concentration according to the HPLC analysis. The results of the sensitivity test showed that the lowest concentration that was still detectable was a dilution factor (DF) of 20, as shown in Figure 4. S7 had a concentration of 262.59 ppm; thus, the lowest concentration of dexamethasone that could be detected was seen at a DF of 20, which was 13.13 ppm. 

#### 3.4.4. Stability Test

**Figure 5:**
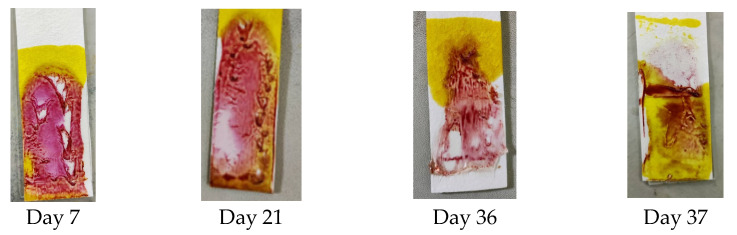
Results of the stability test on the PMMA indicator strip.

A stability test was performed to determine the PMMA-based indicator’s stability and show the resistance of the indicator when detecting dexamethasone at different time intervals after the blending of the reagent. The stability test was carried out every day until the indicator did not provide a color change or produce a positive result. The results shown in Figure 5 indicate that until day 36, the indicator strip still gave an appropriate color change, but on day 37, the indicator’s color did not change to pink-purple, so the stability of the PMMA indicator strip lasted for 36 days. 

## 4. Conclusions

Indicator strips were made to facilitate the process of dexamethasone screening in herbal samples. The indicator strips were made by mixing polymethylmethacrylate (PMMA) with a sulfuric acid reagent by using the reagent blending method. These indicator strips were proven to be able to selectively detect dexamethasone, had stability for up to 36 days, and could detect dexamethasone in herbal samples at concentrations as low as 13.13 ppm. The indicator strips also showed good agreement with TLC and HPLC analyses. In the performance test on real samples, five out of nine samples (S1, S4, S7, S8, and S9) were positive for dexamethasone, which was indicated by an immediate change in the color of the indicator strips from colorless to pink-purple. These indicator strips offer a simple and easy-to-use method that can be carried out on site to detect dexamethasone in herbal samples.

## Figures and Tables

**Table 1:** Legality of the samples according to the website of the Food and Drug Administration of Indonesia.

Sample	Manufacturer	Legality	
		Legal	Illegal
Control (pain, herbal)	PT. A	✓	
S1 (gout, herbal)	PT. B		✓
S2 (pain, herbal)	PT. C	✓	
S3 (pain, herbal)	PT. D		✓
S4 (pain and gout, herbal)	PT. E		✓
S5 (pain, gout, and rheumatic, herbal)	PT. F	✓	
S6 (pain and gout, herbal)	PT. G		✓
S7 (gout and rheumatic, herbal)	PT. H		✓
S8 (pain, herbal)	PT. I		✓
S9 (pain, herbal)	PT. J		✓

**Table 2:** Selectivity test of H_2_SO_4_ with dexamethasone.

Chemical Drug	Result
Dexamethasone	Light pink-purple
Paracetamol	Light brown
Allopurinol	Colorless
Antalgin	Colorless
Phenylbutazone	Colorless
Mefenamic acid	Yellow with a white precipitate
Chlorpheniramine maleate (CTM)	Colorless

**Table 3:** Rf value of each spot and its indication.

Sample	Rf Value	Result
Standard Dexamethasone	0.3 (±0.005)	+
Positive Control	0.285 (±0.008)	+
Negative Control	−	−
S1	0.28 (±0.014)	+
S2	−	−
S3	−	−
S4	0.275 (±0.014)	+
S5	−	−
S6	−	−
S7	0.285 (±0.007)	+
S8	0.295 (±0.007)	+
S9	0.29 (±0.007)	+

Information: (+): containing dexamethasone; (−): not containing dexamethasone.

**Table 4:** Qualitative and quantitative tests for dexamethasone in the herbal samples by using HPLC.

Sample	Rt (min)	AUC	Concentration (ppm)
S1	3.492	4,636,761.3	818.59
S4	3.496	1,741,291.3	297.93
S7	3.502	1,544,784.4	262.59
S8	3.485	2,416,122.5	419.27
S9	3.491	6,032,553.5	1069.58

**Table 5:** Results of the accuracy test with indicators.

Repetition Measurement	Negative Control	Positive Control	S1	S4	S7	S8	S9
1	(−)	(+)	(+)	(+)	(+)	(+)	(+)
2	(−)	(+)	(+)	(+)	(+)	(+)	(+)
3	(−)	(+)	(+)	(+)	(+)	(+)	(+)
Visualization	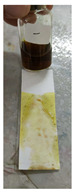	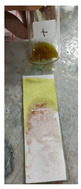	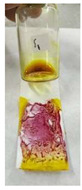	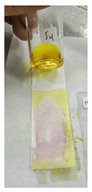	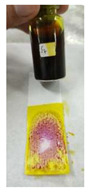	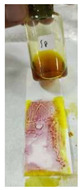	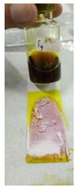

(−) Negative result; (+) positive result.

## Data Availability

Data are available within the article.

## Supplementary Materials

The following are available online at https://www.mdpi.com/article/10.3390/polym15132862/s1, Figure S1: The chromatogram of dexamethasone standard, Figure S2: The chromatogram for each sample that containing dexamethasone.
