# Supplementary material for: A Polymer-Based Indicator for Detecting Dexamethasone in Herbal Medicine Using Polymethylmethacrylate (PMMA)
Source: Polymers (Basel). 2023 Jun 28;15(13):2862. doi: 10.3390/polym15132862 (PMC10346569; doi:10.3390/polym15132862)
Supplement: Supplementary file 1 [file polymers-15-02862-s001.zip › polymers-2433185-supplementary.pdf]

Supplementary Information

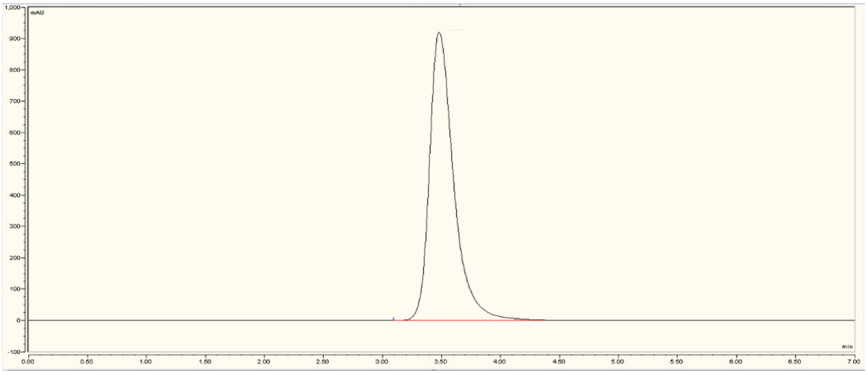

Figure S1. The chromatogram of dexamethasone standard

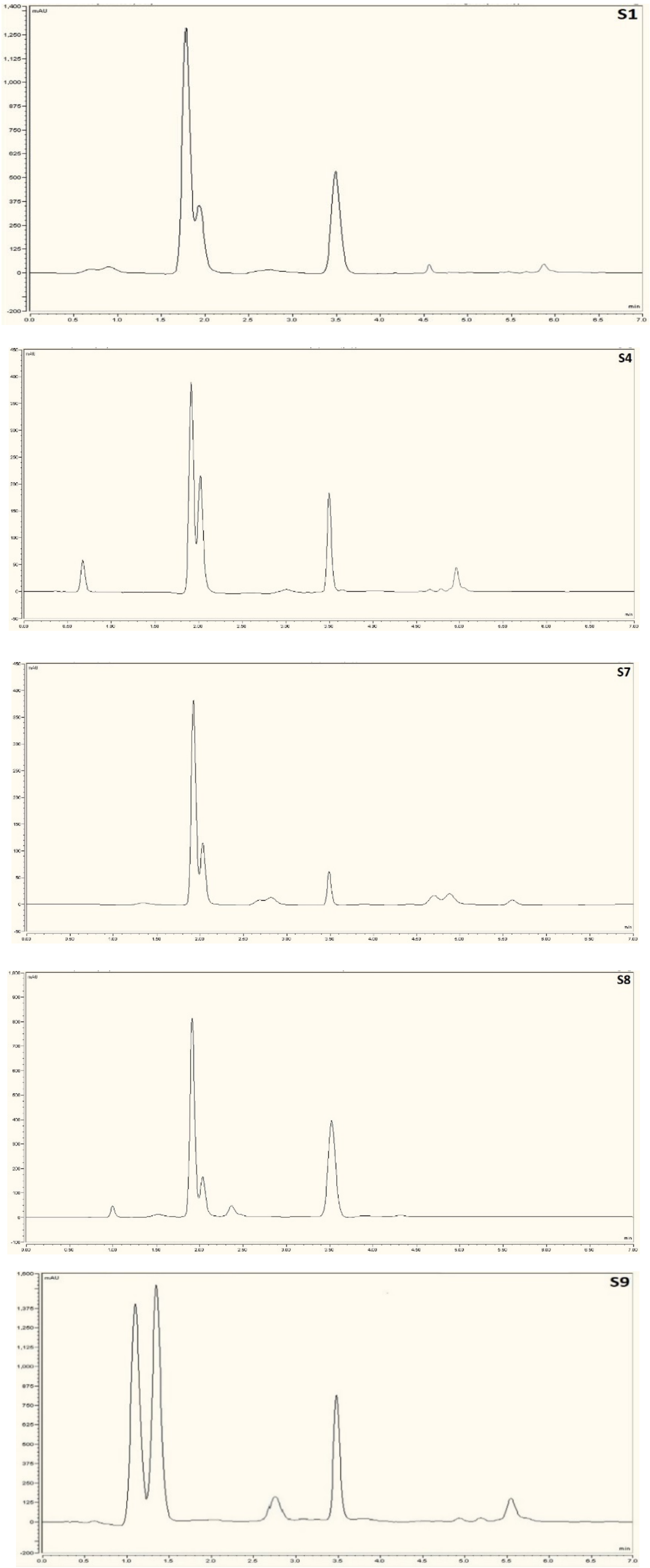

Figure S2. The chromatogram for each sample that containing dexamethasone
